# Feline Infectious Peritonitis mRNA Vaccine Elicits Both Humoral and Cellular Immune Responses in Mice

**DOI:** 10.3390/vaccines12070705

**Published:** 2024-06-24

**Authors:** Terza Brostoff, Hannah P. Savage, Kenneth A. Jackson, Joseph C. Dutra, Justin H. Fontaine, Dennis J. Hartigan-O’Connor, Randy P. Carney, Patricia A. Pesavento

**Affiliations:** 1 Department of Pathology, Microbiology, and Immunology, School of Veterinary Medicine, University of California, Davis, CA 95616, USA; hpsavage@ucdavis.edu (H.P.S.); kajackson@ucdavis.edu (K.A.J.); papesavento@ucdavis.edu (P.A.P.); 2 Department of Medical Microbiology and Immunology, School of Medicine, University of California, Davis, CA 95616, USA; jdutra@ucdavis.edu (J.C.D.); jhfontaine@ucdavis.edu (J.H.F.); dhartigan@ucdavis.edu (D.J.H.-O.); 3 Department of Biomedical Engineering, University of California, Davis, Davis, CA 95616, USA; rcarney@ucdavis.edu

**Keywords:** feline coronavirus, feline infectious peritonitis, nucleocapsid, mRNA vaccine

## Abstract

Feline infectious peritonitis (FIP) is a devastating and often fatal disease caused by feline coronavirus (FCoV). Currently, there is no widely used vaccine for FIP, and many attempts using a variety of platforms have been largely unsuccessful due to the disease’s highly complicated pathogenesis. One such complication is antibody-dependent enhancement (ADE) seen in FIP, which occurs when sub-neutralizing antibody responses to viral *surface* proteins paradoxically enhance disease. A novel vaccine strategy is presented here that can overcome the risk of ADE by instead using a lipid nanoparticle-encapsulated mRNA encoding the transcript for the *internal* structural nucleocapsid (N) FCoV protein. Both wild type and, by introduction of silent mutations, GC content-optimized mRNA vaccines targeting N were developed. mRNA durability in vitro was characterized by quantitative reverse-transcriptase PCR and protein expression by immunofluorescence assay for one week after transfection of cultured feline cells. Both mRNA durability and protein production in vitro were improved with the GC-optimized construct as compared to wild type. Immune responses were assayed by looking at N-specific humoral (by ELISA) and stimulated cytotoxic T cell (by flow cytometry) responses in a proof-of-concept mouse vaccination study. These data together demonstrate that an LNP–mRNA FIP vaccine targeting FCoV N is stable in vitro, capable of eliciting an immune response in mice, and provides justification for beginning safety and efficacy trials in cats.

## 1. Introduction

Feline infectious peritonitis (FIP) is a fatal disease caused by feline coronavirus (FCoV). FCoV is an enveloped, positive-sense, single-stranded RNA virus in the Alphacoronavirus genus. Taxonomically, FCoV is classified as species Alphacoronavirus 1, along with canine coronavirus and transmissible gastroenteritis virus of pigs [1,2,3,4,5,6,7,8]. These coronaviruses share similar biological features, including high transmissibility and prevalence, frequent recombination events, potential for persistence, and potential to cause significant disease in their respective hosts [2,5,9,10,11,12,13,14]. Estimates of FCoV seroprevalence reach 87% in cats living in high density environments such as shelters and catteries [1,15,16,17]. In these environments where FCoV is endemic, as many as 5–10% of cats may develop one of a spectrum of viral mutations that cause fatal systemic FIP [18,19]. Despite the large burden of infection and disease, all attempts thus far to generate a safe and effective vaccine to prevent the development of FIP have failed [20,21].

Fundamentally, the reasons for FIP vaccine failure include a remarkably complicated host–virus relationship and gaps in our understanding of disease pathogenesis and immune correlates of protection. There are two “serotypes” of FCoV, with type 1 predominating worldwide [21,22,23]. Serotype 2 is the result of a recombination event of type 1 FCoV with the closely related canine coronavirus spike (S) gene, therefore, no cross-protection between serotypes from an immune response mounted towards S would be expected.

Upon initial infection, which typically occurs in very young kittens, the enteric form of FCoV replicates in intestinal epithelium where fecal shedding may persist for many months [21,24,25]. According to the generally accepted “internal mutation” theory, an unpredictable subset of these infected cats may generate viruses that undergo one of a number of mutations or deletions, each of which are considered a switch to the FIP virus (FIPV) [4,26,27,28]. These genetic changes are associated with altered viral tropism from intestinal epithelium to monocytes/macrophages, resulting in widespread viral dissemination, vasculitic multi-organ granulomatous disease, and death.

Because there are multiple genetic mutations that can define the FCoV to FIPV switch, most of which occur in the spike (S) gene, vaccines targeting single FIPV protein epitopes or targeting only one of the two known serotypes of FCoV would only protect individual cats harboring specific mutations or with infections from a specific serotype. Moreover, an antibody response to S has been experimentally shown to elicit paradoxical worsening of disease upon subsequent exposure to the virus [29,30,31,32]. This phenomenon is known as antibody-dependent enhancement (ADE) of infection. ADE occurs when non-neutralizing or sub-neutralizing concentrations of antibodies bind to the viral surface, mediating uptake into monocytes and macrophages through Fc-receptor binding. Under normal conditions, this would help eliminate the pathogen and disease; however, FIPV replicates very efficiently in those cells, resulting in viral dissemination and augmented disease [21,33,34]. For these reasons, a vaccine strategy targeting S carries with it risks of both inefficacy and, importantly, significant safety concerns.

As an alternative, several groups have examined using the internally expressed nucleocapsid (N) protein as a vaccine target for FIP [35,36,37]. The principle behind using this target is that, rather than inducing sterilizing immunity, eliciting a robust CD8+ T cell response to N will mediate clearance of infected cells. N is genetically highly conserved across both serotypes of virus, making it an excellent vaccine target. Additionally, experimental studies have demonstrated that a robust CD8+ T-cell-focused immune response to N may be protective and aid in the clearance of FCoV [38]. This study describes the development of a lipid nanoparticle (LNP)-encapsulated mRNA vaccine targeting FCoV N to prevent FIP. Both in vitro and preliminary in vivo proof-of-principle studies are presented suggesting that this vaccine is an excellent candidate to prevent FIP in cats.

## 2. Materials and Methods

### 2.1. Cell Culture and Transfection

Crandell–Rees feline kidney (CRFK; ATCC #CCL-94) cells were grown in minimal essential medium with Earle’s balanced salts, supplemented with 10% fetal bovine serum, 100 units/mL penicillin, and 100 µg/mL streptomycin, 1 mM sodium pyruvate, and 1× non-essential amino acids, and incubated at 37 °C and 5% CO_2_ in a humidified incubator. All cell culture reagents were purchased from Gibco/Thermo Scientific (Waltham, MA, USA).

For quantitative reverse-transcriptase PCR (q-RT PCR), cells were transfected in 24-well plates in biological triplicate for each construct and timepoint, using 500 ng of mRNA per 90% confluent well (Lipofectamine 3000, Invitrogen, Waltham, MA, USA). For immunofluorescence assay (IFA), two wells per construct and timepoint were used to transfect eight well chamber slides with 250 ng of mRNA per 90% confluent well. For downstream mRNA q-RT PCR, cells from each of 3 wells per construct were harvested at day 0, 1, 2, 3, 5, and 7 post-transfection (day 0 cells were washed 3 times then harvested immediately post-transfection). For downstream immunofluorescence assay, cells were washed 3 times, then fixed in 4% paraformaldehyde for 25 min at room temperature, then rinsed in phosphate-buffered saline (PBS) and stored at 4 °C until time of downstream analysis.

### 2.2. In Vitro Transcription and mRNA Purification

Sequence from a circulating strain of FCoV (Genbank KF530271.1) was used as a basis for nucleocapsid constructs. Plasmids encoding silent mutations to optimize GC content (with preferential use of common feline codons) and wild type (WT) nucleocapsid were designed in-house and synthesized commercially (GenScript, Piscataway, NJ, USA). Additional modifications for optimized mRNA stability and protein expression were made as described by others [39,40,41]. Linearized plasmid or amplified PCR product was transcribed in vitro (HiScribe T7, NEB, Ipswich, MA, USA), with co-transcriptional capping (CleanCap, TriLink BioTechnologies, San Diego, CA, USA) and using N^1^-Methylpseudouridine (TriLink BioTechnologies) per manufacturer’s protocols. Uncapped mRNA for each construct with CleanCap eliminated from the transcription reaction was used as a control for in vitro studies. mRNA was column-purified (Monarch RNA, NEB) then subjected to cellulose purification per previously published protocol and stored at −80 °C until further use [42]. Quality control was performed using a 2100 BioAnalyzer RNA Nano Assay (Agilent, Santa Clara, CA, USA), with no contamination seen (DNA Technologies and Expression Analysis Core, UC Davis) (Figure S3).

### 2.3. Measurement of Nucleocapsid mRNA Stability by Quantitative Reverse-Transcriptase PCR (q-RT PCR)

At each timepoint indicated, q-RT PCR was performed on RNA purified from transfected cells. Two primer sets were tested, comparing primer efficiency on a standard curve using diluted plasmid with copy number quantified. The primer set with highest efficiency was chosen for use (Figure S1). Known copy number of diluted plasmid was included with each run for quality control. Assay was performed with technical triplicates on each of 3 biological replicates per construct and timepoint; results are the average of the 9 replicates per construct and timepoint. Cells were washed 3 times in PBS, trypsinized, and harvested. RNA from each well was extracted separately (RNEasy, Qiagen, Hilden, Germany). Extracted RNA was subjected to reverse transcription (QuantiTect Reverse Transcription, Qiagen) and used for qPCR using primers FCoV N.359F CCATGAACAAGCCAACGACACT and FCoV N.464R CGGTTCACTTCAAGCTGGAATTG, amplifying a 106 bp region of the gene (Maxima SYBR Green, Thermo Fisher, Waltham, MA, USA). Limit of detection = 50 copies/reaction.

### 2.4. Immunofluorescence Assay (IFA)

Fixed cells were permeabilized for 10 min in 0.5% sodium deoxycholate in PBS. Cells were then blocked in 5% normal goat serum in PBS + 0.1% Triton-X (PBS-T) for 1 h at room temperature. Primary monoclonal mouse anti-feline nucleocapsid (Bio-Rad, Hercules, CA, USA) was used at 1:1000 in PBS-T for 1 h at room temperature. Cells were washed 5 times in PBS-T, then secondary goat anti-mouse AlexaFluor488 (Invitrogen) was used at 1:1000 for 20 min at room temperature; cells were counterstained with DAPI at 300 nM for the last 5 min of secondary staining. Cells were washed 5 times in PBS-T, and left in PBS-T for imaging. Imaging was performed on an EVOS AMG digital inverted microscope (Life Technologies, Carlsbad, CA, USA), and images were captured with equal intensity, brightness, and contrast. Approximately 10–12 20× representative images per well in areas of ~80% cell confluence were captured for ImageJ fluorescence quantification normalized to day 0 and uncapped transfected controls.

### 2.5. Western Blot

CRFK cells at 1 day post-transfection were lysed in RIPA lysis buffer containing protease inhibitor cocktail (Roche, Basel, Switzerland). Equivalent cell number was loaded on a 4–12% gradient Tris–glycine SDS–polyacrylamide gel and proteins were transferred to a 0.2 μm PVDF membrane (Life Technologies). Membranes were blocked in 5% milk in PBS with 0.05% Tween-20 for one hour at room temperature with rocking, and then incubated with primary mouse anti-feline nucleocapsid antibody in blocking buffer at 4 °C overnight at a dilution of 1:1000 (BioRad). Secondary anti-mouse IgGκ-HRP was diluted at 1:5000 in block buffer and incubated at 1 h at room temperature with rocking (Santa Cruz Biotechnology, Dallas, TX, USA). Proteins were visualized with Supersignal West Pico PLUS chemiluminescent substrate (ThermoFisher) using FluorChem E (Protein Simple, San Jose, CA, USA).

### 2.6. LNP Encapsulation

LNPs were derived using a working concentration of 0.17 mg/mL diluted in formulation buffer (Precision NanoSystems, Vancouver, BC, Canada) and GenVoy-ILM^TM^ at a flow rate ratio of 3:1. Organic and aqueous solutions were used at N/P ratios of 5 or 6 for optimization. Solutions were loaded in cartridges on the NanoAssemblr^®^ Ignite^TM^ apparatus and resulting LNPs were buffer exchanged in PBS on Centricon centrifugal devices per manufacturer’s instructions (10 kDa NMWL, Millipore, Burlington, MA, USA).

Recovered LNP–mRNA size and dispersity was characterized by dynamic light scattering (Malvern Zetasizer Nano ZS, Zetasizer Software version 7.11), size and quantity characterized by nanoparticle tracking analysis, and encapsulation efficiency and concentration determined by RiboGreen RNA Assay Kit (Invitrogen) per previously described protocols provided by Precision NanoSystems. For nanoparticle tracking analysis (NTA), mRNA-encapsulated LNPs were diluted in 0.2 μm filtered PBS to a final concentration between 1 × 10^7^ and 2 × 10^9^ particles/mL and loaded by syringe pump (Harvard Bioscience, Holliston, MA, USA). The NanoSight LM10 (Malvern Panalytical Ltd., Westborough, MA, USA) was used for data collection with NanoSight NTA 3.1. software for analysis. Three 90 s videos were collected to determine an average concentration and size profile of particles with camera level of 10 and detection threshold of 2. Between samples, MilliQ water was used to clear out the sample lines.

### 2.7. Mouse Vaccination and Safety Assays

This study followed the ethical guidelines and was approved by UCD IACUC (protocol number 21796). Ten 12-week-old C57BL/6J mice were bled prior to vaccination, then vaccinated with 10 µg of WT (n = 4), GC-optimized (n = 4), or mock-vaccinated with PBS at weeks 0 and 6 by the subcutaneous route in the left pelvic limb. No adverse events were noted in mice after either prime or boost. Mice were euthanized at 5 weeks post-boost and terminally bled at the time of euthanasia. Splenocytes were harvested for flow cytometry, and serum spun for antibody titers and stored at −20 °C until time of use by enzyme-linked immunosorbent assay (ELISA).

### 2.8. IgG-Specific Nucleoprotein Antigen ELISA

Next, 96-well plates were coated with purified recombinant feline coronavirus nucleocapsid protein at 5 µg/mL overnight at 4 °C (ICL, Inc., Portland, OR, USA). Plates were blocked with 1% bovine serum albumin in PBS for 1 h at 37 °C. Mouse serum was added in twofold serial dilution in PBS and performed in technical duplicates, and positive control antibody (mouse anti-FCoV monoclonal, BioRad) was used at 1:1000. Plates were incubated for 2 h at room temperature, then washed 4× in PBS. Secondary anti-mouse IgGκ-HRP (Santa Cruz Biotechnology) was diluted at 1:5000 and added for 1 h at room temperature, then washed 4× in PBS. Detection with tetramethylbenzadine substrate and peroxide solution, with reaction stopped after ~4 min with 2 M sulfuric acid, and plates were read for absorbance at 450 nm. For each twofold dilution, technical duplicates of two representative negative samples (pooled pre-bled mouse serum from all 10 mice and pooled terminal bleed from PBS mock-vaccinated mice) were used to determine negative titers.

### 2.9. Splenocyte Stimulation Assay

After necropsy, spleens were homogenized by passing through a 40 µm cell strainer. Red blood cells were lysed with ACK lysing solution (Gibco), washed and resuspended in RPMI media containing 2 mM L-Glutamine, 5% serum, 1× non-essential amino acids, 1 mM sodium pyruvate, 100 units/mL penicillin, and 100 µg/mL streptomycin.

Splenocytes were plated at 2 × 10^6^ cells/well in 96-well U-bottom plates and stimulated with 2 µg/mL of a 15 m overlapping peptide pool spanning the entire FCoV N protein (Intavis). After two hours, golgi plug protein transport inhibitor (BD Biosciences, San Diego, CA, USA) was added and cells were incubated overnight at 37 °C 5% CO_2_ in a humidified incubator. Positive stimulation control, using cell activation cocktail containing phorbol-12-myristate 13-acetate/ionomycin, was performed according to the manufacturer’s protocol (BioLegend, San Diego, CA, USA). Unstimulated controls were treated with 0.5% DMSO. Cell counts were obtained using a TC20 automated cell counter (BioRad). Following stimulation, the cells were stained, fixed, and acquired the same day.

### 2.10. Flow Cytometry

Fc receptor blocking was performed using TruStain FcX PLUS anti-mouse CD16/32 antibody (BioLegend) and surface antigen staining was performed at 4 °C for 30 min using the following antibodies: APC/Fire 750 anti-mouse CD3ε 145-2C11 (BioLegend, 1:50), AF 488 anti-mouse CD4 GK1.5 (BioLegend, 1:100), APC-R700 Rat Anti-Mouse CD8a 53-6.7 (BD Biosciences, 1:200). Dead cells were identified using Zombie Aqua Fixable Viability dye (BioLegend). Following surface staining the cells were fixed and permeabilized using the Cytofix/Cytoperm Plus Fixation/Permeabilization Kit (BD Biosciences) for 20 min at 4 °C. Intracellular cytokine staining was performed using antibodies PE anti-mouse IL-2 JES6-5H4 (BioLegend, 1:100), APC anti-mouse interferon gamma (IFN-γ) XMG1.2 (BioLegend, 1:100) and PE/Cyanine7 anti-mouse tumor necrosis factor alpha (TNF-α) MP6-XT22 (BioLegend, 1:100) in BD Perm/Wash buffer at 4 °C for 30 min.

Sample fluorescence and cell characteristics were assessed using a Beckman Coulter Cytoflex S 4-laser (Brea, CA, USA), 13-color flow cytometer with CytExpert software v. 2.6. Compensation, gating and analysis were performed using FlowJo 10.9.0 (Ashland, OR, USA).

### 2.11. Data Analysis and Statistics

All data were analyzed in Graph Pad Prism (10.2.1, Boston, MA, USA). Unless otherwise indicated, all results represent two-sample t-tests. Serum ELISA titers were determined by previously described methods for determining endpoint titers using a 95% confidence interval based on two negative controls as described above [43].

## 3. Results

### 3.1. Vaccine Design

**Figure 1:**
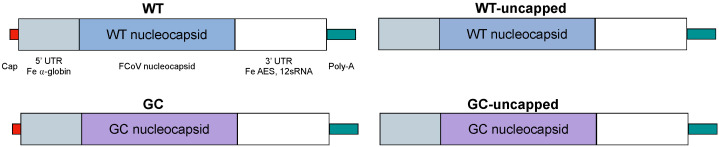
mRNA vaccine sequence and design. Modifications commonly applied to the 5′ and 3′ UTRs were used for both WT mRNA (“WT”) and GC-content optimized mRNA (“GC”). For in vitro studies, identical sequences were used but co-transcriptional capping was not performed as an mRNA control without the presence of protein production.

Forty sequences from Genbank published within the last 20 years from locations spanning the globe were compared, and one sequence representing approximately 90% identity across the N genes in this subset was chosen as wild type (WT) template (Genbank accession # KF530271) (Figure S2). This WT sequence contains a GC content of 44.4%. Silent mutations were introduced to increase GC content to 57.6%, with preferential use of common codons in the cat; however, codon optimization was not a primary goal, as the wild-type virus has been in circulation in the feline population for at least six decades. Modifications previously described to increase mRNA stability and protein production were made to the 5′ and 3′UTR and the poly-A tail, with cat-specific modifications introduced where appropriate [39,40,41]. These mRNAs and their resulting LNP-encapsulated vaccine constructs are referred to throughout as “WT” and “GC” (Figure 1). Uncapped mRNA constructs were derived as controls for in vitro experiments, which are identical in nature to their capped counterparts except that the co-transcriptional inclusion of a 5′ cap was omitted (and, therefore, cannot be translated). These constructs are referred to as “WT-uncapped” and “GC-uncapped”.

### 3.2. mRNA Stability and Expression In Vitro

**Figure 2:**
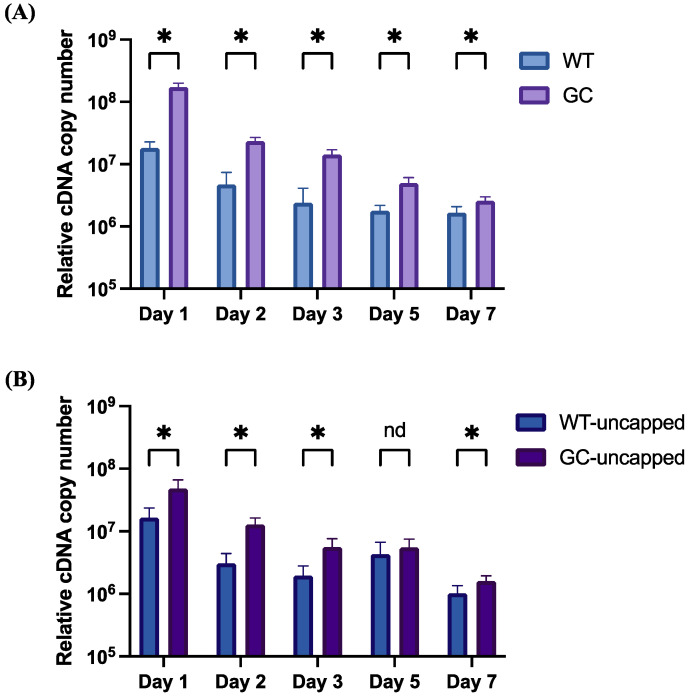
Relative mRNA abundance after in vitro transfection with FCoV N mRNA. Equivalent cell numbers were extracted for q-RT PCR at the indicated timepoints post transfection. Plotted are mean and standard deviation of relative cDNA copy number at each time point, with averages representing biological triplicates and qPCR run in technical triplicates per sample. (**A**) Comparison of capped WT vs. GC mRNA; (**B**) comparison of uncapped WT vs. GC mRNA. For comparisons, * = *p* < 0.05, nd = no difference.

Cultured Crandell–Rees feline kidney (CRFK) cells were transfected with purified mRNA and assayed for mRNA stability by quantitative reverse-transcriptase PCR (q-RT PCR) at days 1, 2, 3, 5, and 7 post-transfection (Figure 2). mRNA quantity peaked at day 1 and decreased over the course of one week, remaining detectable at all timepoints tested. Both capped and uncapped GC constructs demonstrated significantly higher quantities of mRNA as compared to their respective WT constructs at all timepoints except for one (uncapped, day 5) where no difference was seen between the two. The rate of decay decreased over time with both pairs of constructs, with the biggest difference in means between each pair occurring at day 1. Overall, these experiments demonstrate increased stability of GC mRNA in cultured feline cells as compared to wild type.

### 3.3. Protein Production In Vitro

**Figure 3:**
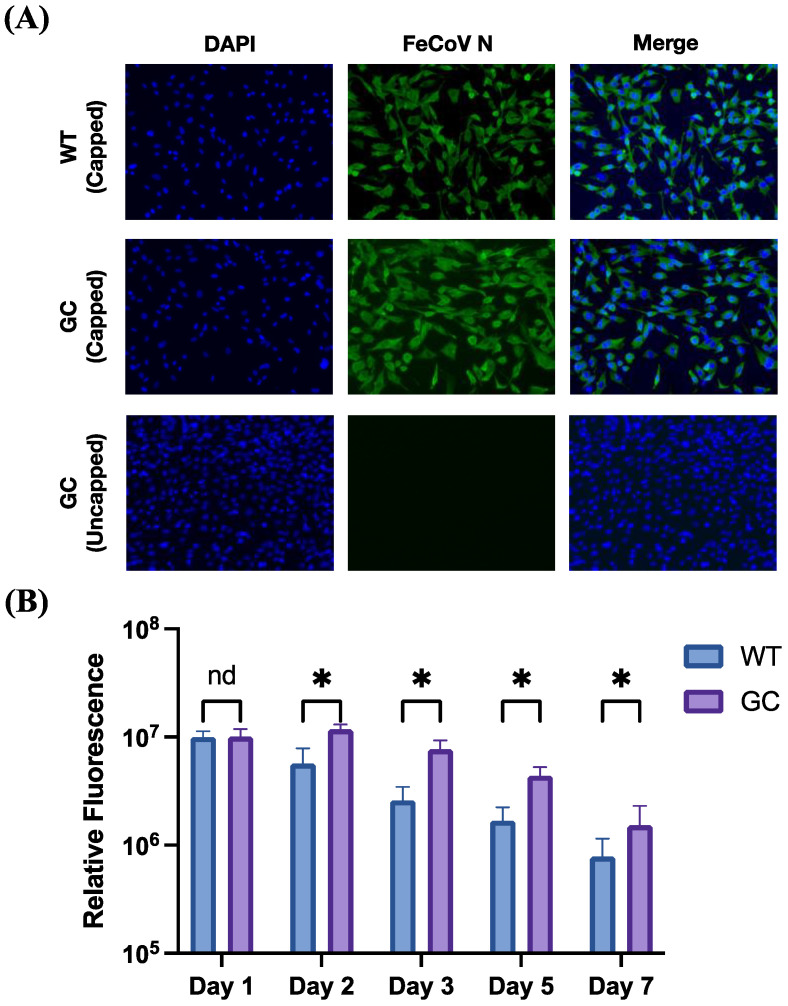

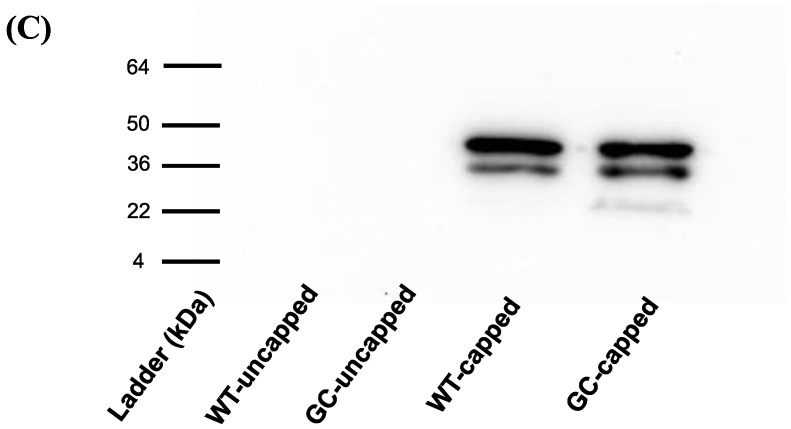
Relative protein expression by IFA after in vitro transfection with FCoV N mRNA. (**A**) Representative images of FCoV N expression (green, middle and right panels) at 1 day post-transfection. Nuclei are stained with DAPI in blue; top panels represent transfection with capped WT mRNA, middle panels represent transfection with capped GC mRNA, and bottom panels represent transfection with uncapped GC mRNA (negative control). Images have been enhanced identically in this figure. (**B**) Integrated density was measured at the time points indicated, thresholded to mock-transfected and day 0 transfected average. Plotted are mean and standard deviation of ~10 10× images at each time point. For comparisons, * = *p* ≤ 0.02, nd = no difference. (**C**) Western blot was run for cells transfected with indicated mRNAs at day 1 post-transfection, with equivalent cell numbers loaded in each lane (expected size ~50 kDa).

Post-transcriptional control of translation can affect the total amount of antigen produced by transfected cells; therefore, quantification of protein by immunofluorescence assay (IFA) was performed. CRFKs were transfected and fixed at the same timepoints as in Section 3.2 then stained by indirect IFA. Multiple images were taken from each construct/timepoint from each transfected well, and protein was quantified with ImageJ by measuring the integrated density of pixels from these images. Uncapped and day 0 (fixed immediately post-transfection) wells were used for thresholding; no overt fluorescence from N was visually noted in any of these samples. Representative images of day 1 post-transfection show robust protein expression in both WT and GC constructs (Figure 3A,C). Quantitatively, day 1 was the only time point where no significant difference was seen between WT and GC constructs; all other timepoints demonstrated significantly increased protein from the GC construct, with protein expression persisting but declining through day 7 post-transfection (Figure 3B; *p* < 0.0001 for days 2–5, *p* = 0.02 for day 7).

Finally, to characterize the size of the protein expressed, Western blot was performed on cells at 1 day post-transfection, and correct size was confirmed (Figure 3C).

These experiments together confirm protein production to closely follow mRNA quantity in vitro in feline cells, with increased protein produced from GC-transfected cells as compared to WT.

### 3.4. Mouse Vaccination and Humoral Immune Response

Vaccine constructs were characterized by size and dispersion using dynamic light scattering and nanoparticle tracking analysis, and for encapsulation efficiency by Ribogreen assay (Table 1). Based on particle size and encapsulation efficiency, optimal N/P ratio constructs were selected for in vivo studies (WT—N/P 5, GC—N/P 6).

**Figure 4:**
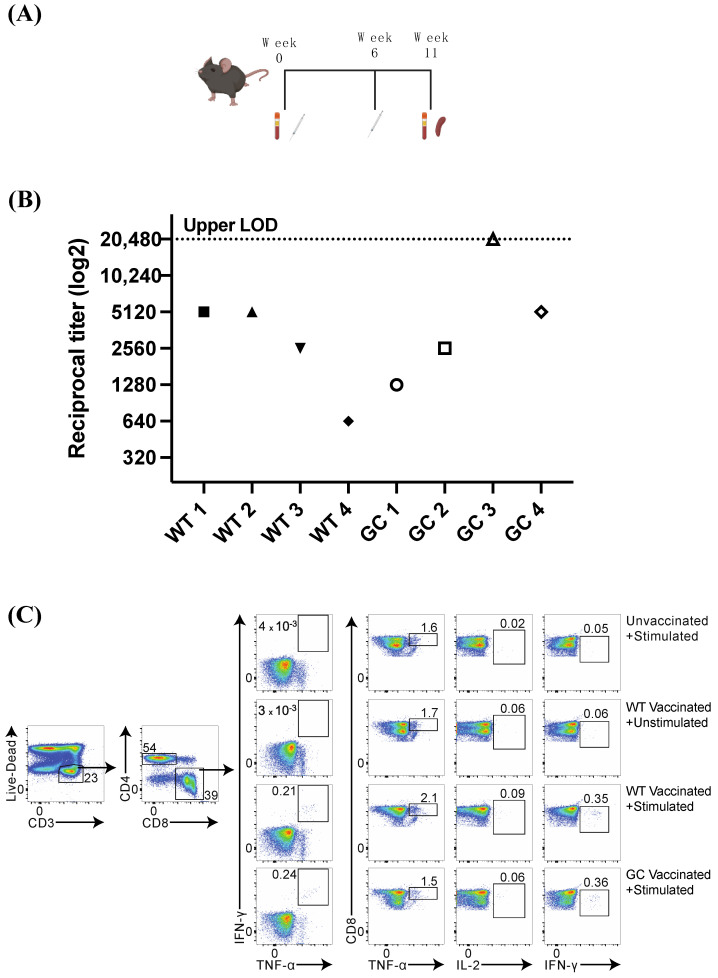
In vivo immune responses in mice. (**A**) Schematic of mouse study; 10 mice were bled before vaccination (week 0), then vaccinated with WT (n = 4), GC (n = 4), or mock-vaccinated with PBS (n = 2). Mice were boosted at week 6 with the same vaccine or PBS, then euthanized at week 11 for serum and spleen collection. (**B**) Endpoint serum antibody titers were measured by ELISA and plotted as reciprocal titers, with each mouse represented along the X axis (WT 1–4 represent each of the four mice vaccinated with WT; GC 1–4 represent each of the four mice vaccinated with GC) (95% confidence interval). (**C**) Endpoint splenocytes were stimulated overnight with overlapping peptides corresponding to the entire N protein, then analyzed by flow cytometry. Gating strategy and representative plots are shown for each group. Unvaccinated + Stimulated = PBS (mock)-vaccinated mouse splenocytes stimulated overnight with peptide pool; WT Vaccinated + Unstimulated or Stimulated = WT-vaccinated mouse splenocytes stimulated overnight with 0.5% DMSO (Unstimulated) or with peptide pool (Stimulated); and GC Vaccinated + Stimulated = GC-vaccinated mouse splenocytes stimulated overnight with peptide pool. Markers of stimulation and immune activation (TNF-α, IL-2, and IFN-γ +) are shown as percentages of Live CD3+CD8+ cells.

For this, 12-week-old C57BL/6J mice were vaccinated with a prime boost strategy at weeks 0 and 6 (Figure 4A). Four mice per vaccine construct were used, with two PBS mock-vaccinated controls. Blood was collected prior to vaccination and again at euthanasia (week 11) for N-specific IgG quantification by ELISA. All eight vaccinated mice developed antibody responses at the time of euthanasia, with endpoint titers ranging from 1:640 to 1:5120 (WT) and 1:1280 to the upper limit of detection at 1:20,480 (GC) (Figure 4B). Titers from PBS-vaccinated (pre and post) and pre-vaccinated (all) pooled serum were undetectable.

### 3.5. Nucleocapsid-Specific CD8+ T Cell Response

Splenocytes were harvested at the time of euthanasia (week 11) and stimulated with FCoV N overlapping peptides to detect antigen-specific T cell responses. Flow cytometry plots from representative mice in described vaccinated groups are presented in Figure 4C, with details from individual mice presented in Table 2. After stimulation and compared to PBS controls, most mice developed N-specific CD8+ T cells expressing interferon gamma (IFN-γ), with a range in WT-vaccinated mice from 0.17 to 1.7% of total CD8+ T cells and a range in GC-vaccinated mice from 0.14 to 0.54% as compared to PBS (mock-vaccinated) control mice (average 0.096%) (Table 2). CD8+ T cells which were double-positive for TNF-α and IFN-γ were present at a range of 0.1 to 1.6% of WT-vaccinated mice, and a range of 0.05 to 0.39% of GC-vaccinated mice, as compared to PBS controls (average 0.01%). All vaccinated mice in both groups had higher percentages of TNF-α and IFN-γ double-positive CD8+ T cells after peptide stimulation compared to PBS controls, indicating that an effective N-specific CD8+ T cell immune response was elicited after vaccination.

Finally, splenocytes were analyzed for nucleocapsid mRNA by q-RT PCR after euthanasia; all results were negative (C_t_ above limit of detection).

## 4. Discussion

FIP remains one of the highest-burden fatal infectious diseases in cats. Viruses with the potential to cause ADE have been notoriously difficult to vaccinate for due to the potential risk of vaccination worsening patient outcomes. The only available FIP vaccine for use in the United States is limited by the range of virus it protects from (serotype 2) and the minimum age at which it is labeled to be administered to prevent initial FCoV infection (16 weeks, by which time most cats are already FCoV positive) [30,44,45].

Many diseases that have been historically recalcitrant to successful vaccine development are now being reconsidered due to the rapidly expanding field of LNP-encapsulated mRNA vaccines [46,47]. An effective vaccine strategy to protect cats from FIP should not only have the potential to limit spread after an initial FCoV infection but should also help a persistently infected cat clear the virus prior to the onset of FIP. The strategy described here abrogates concerns of ADE by targeting N, which is internal to the viral envelope, and has the potential to elicit a robust cell-mediated immune response, which could help clear either a novel or a previously established FCoV infection to preclude the development of FIP.

This study represents the first of its kind in developing an mRNA vaccine for FIP. This platform has heretofore not been widely studied in veterinary medicine, and some of the unique features inherent to mRNA vaccines may overcome many of these historical challenges. mRNA vaccines provide many distinct advantages over more traditional vaccine platforms, including a very high safety profile, induction of intracellular antigen production to more closely mimic natural infection, and ever-increasing ease of development and adaptation to new and emerging pathogens. Of particular importance in the cat, the LNP itself serves as the adjuvant, negating the need for more traditional adjuvants that can be associated with the development of feline injection site sarcomas. Additionally, increasing epidemiologic evidence is emerging to suggest that the initial series of mRNA vaccination can yield a robust and long-lasting memory T and B cell response [48,49,50,51]. Taken together, these properties make mRNA vaccines a highly promising strategy to address infectious diseases for which no safe and effective vaccine exists in veterinary medicine.

Of note, there is one USDA-approved vaccine for FIP in cats [44]. Several other vaccines have been attempted, but none have reached the market for use. The single approved vaccine has seen limited use for multiple reasons, including concerns for safety, lack of cross-protection between the more common serotype (I) and the vaccine serotype (II), and the age at which vaccination, which is meant to *prevent* initial infection, is labeled for use (16 weeks), by which point the majority of cats have already been exposed and likely infected [52,53,54]. This has led to a formal advisory panel recommendation by the American Association of Feline Practitioners that “[at] this time, there is insufficient evidence that the vaccine induces clinically relevant protection, and use of the vaccine is not recommended” [55].

Other mRNA vaccines have been explored for veterinary use, albeit none have been tested for feline diseases, and no mRNA vaccine studies in cats has been described at the time of this publication. Of note, recent FCoV vaccinations using different platforms have proven effective by variable measures. One such study uses an adenovirus-based N vaccine, which elicits a decrease in clinical signs in cats after challenge [37]. However, only one cat in the control group succumbed to challenge, so protection from fatal disease could not be readily assessed. Another limitation of this study is that vaccine was delivered intramuscularly, which would be incongruous with current standard veterinary practice. Additionally, murine studies only examined total numbers of CD4+, CD8+, and CD19+ cells, and did not look for N-specific activation-induced markers. In the feline studies, serum cytokine and antibody levels were measured post-vaccination, but lymphocyte assays were not performed directly.

Another recent FCoV vaccine using the recombinant heptad repeat 2 domain displayed on *Bacillus subtilis* spores [56]. The biggest limitations of this vaccine include the use of peptides within the spike protein of serotype II of FCoV, which, again, is not the predominant circulating type, and the premise of this vaccine to induce sterilizing immunity, which would require vaccination prior to infection. This is the same limitation as seen with the currently approved temperature-sensitive attenuated vaccine available in the US, and vaccination prior to infection for FCoV is an impractical if not impossible goal.

A slightly more distant but mRNA vaccine-based veterinary example targeted porcine epidemic diarrhea virus (PEDV) [57]. PEDV is an enteric coronavirus of pigs and causes significant morbidity and mortality in piglets, representing significant losses in the pig industry [58]. This study demonstrated vaccine efficacy against challenge with PEDV in piglets, with immunogenicity also demonstrated in mice. The focus of this study was also on antibodies, both total virus-specific IgG and neutralizing antibodies from mice and pigs. Again, the virus-specific CD8+ lymphocyte response was not well characterized in this study, as the focus was primarily on antibody production and protection from challenge. Additionally, this is a spike-based vaccine, as ADE is not described in the pathogenesis of PEDV. These are not limitations for this particular disease, as neutralizing antibodies to spike are indeed protective, but limits direct comparison with the mRNA N-directed vaccine for FIP.

While the vaccine described here for FIP is highly promising, several limitations remain in these studies. The most important of these limitations is the use of a pilot-sized in vivo trial in mice. This study demonstrated immunogenicity of the vaccines in all mice, but with a wide range of the level of response. While highly variable, every vaccinated mouse did develop a response higher than the average of mock-vaccinated mice with regards to both N-specific CD8+ T lymphocytes and humoral response. Some of the potential causes of this high variability, beyond normal inter-animal variation (which is more pronounced in a small-scale study), include mRNA instability in the mouse, suboptimal codon usage in mice for these constructs (which have evolved in or were altered specifically for the cat), and the subcutaneous route of administration. A small sample size was chosen because this study was primarily designed as proof-of-concept, and not to achieve statistical significance in a species for which the vaccine is not ultimately intended. The subcutaneous route is typically used for vaccines in veterinary medicine, although results can be more variable than intramuscular administration. Notably, the differences seen between WT and GC constructs are primarily in the in vitro studies performed on cells from the target species. This combined with variable but present immunogenicity in mice warrant next step studies to assess safety and immunogenicity in cats.

Overall, mRNA vaccines have yet to make it to the veterinary market. One of the potential reasons for this include concerns over cost of production and cost-effectiveness to produce an mRNA vaccine for FIP. A recent cost analysis breakdown for the human COVID-19 vaccines estimates that the cost per dose (ignoring cost of research, development, and clinical trials) comes out in the realm of ~USD 2 per dose [59]. This figure would be difficult to directly compare to a potential FIP mRNA vaccine, as the variables at this point of vaccine production for veterinary use are far too complex to accurately assess at this time. Regardless, a cost analysis would have to be performed prior to producing this vaccine at scale for the veterinary market.

mRNA vaccines are at the early stages of development for the veterinary market. A protective FIP vaccine for cats remains a critical need in veterinary medicine. The field of antiviral therapy is rapidly progressing, but little progress in the field of vaccine development has been seen in the past few decades. An LNP-encapsulated mRNA vaccine against FCoV N has demonstrated potential to induce an immune response in animals, and these findings warrant safety and immunogenicity studies in cats.

## 5. Conclusions

This study describing initial development and preliminary proof of concept for a vaccine for FIP found that a CG-content optimized LNP-encapsulated mRNA vaccine targeting N is maintained in feline cells with protein production for at least 1 week after transfection in vitro. This vaccine also elicited both humoral and cellular responses specific to N after a prime-boost vaccination strategy in mice. While the study in mice demonstrated high variability in immune response, no safety concerns emerged, and each individual mouse did mount a specific response to the vaccine. The study here provides a foundation to justify moving forward with in vivo safety and immunogenicity studies in the target feline species.

## 6. Patents

The technology described herein is the subject of a pending U.S. provisional patent application.

## Figures and Tables

**Table 1:** Characterization of WT and GC nucleocapsid mRNA-encapsulated LNP vaccines.

Vaccine	N/P Ratio	Average Diameter DLS (nm)	Polydispersity Index DLS	Average Diameter NTA (nm)	Encapsulation Efficiency Ribogreen (%)
WT	**5**	**96.8**	**0.199**	**100.5**	**96.3**
	6	86.6	0.173	94.7	94.6
GC	5	122.8	0.174	96.7	93.9
	**6**	**117.8**	**0.186**	**95.9**	**96.8**

**Table 2:** Mean percentage of CD8+ T cells expressing cytokines after stimulation in vaccinated mice.

Vaccine–Mouse	IFN-γ	TNF-α	IL-2	TNF-α + IFN-γ
WT-1	0.31	1.94	0.07	0.18
WT-2	1.78	3.22	0.2	1.59
WT-3	0.35	1.27	0.05	0.23
WT-4	0.17	1.50	0.06	0.10
GC-1	0.14	1.71	0.03	0.06
GC-2	0.31	1.25	0.06	0.19
GC-3	0.54	3.72	0.04	0.39
GC-4	0.18	1.32	0.05	0.09
PBS-1	0.05	1.64	0.02	0.004
PBS-2	0.14	2.47	0.02	0.016

## Data Availability

The data presented in this study are available on request from the corresponding author due to pending provisional patent application.

## Supplementary Materials

The following supporting information can be downloaded at: https://www.mdpi.com/article/10.3390/vaccines12070705/s1, Figure S1: qPCR assay validation. Figure S2: mRNA sequence comparison. Figure S3. Bioanalyzer quality control from purified mRNA.
