# Supplementary material for: Feline Infectious Peritonitis mRNA Vaccine Elicits Both Humoral and Cellular Immune Responses in Mice
Source: Vaccines (Basel). 2024 Jun 24;12(7):705. doi: 10.3390/vaccines12070705 (PMC11281389; doi:10.3390/vaccines12070705)
Supplement: Supplementary file 1 [file vaccines-12-00705-s001.zip › vaccines-2997774-supplementary.pdf]

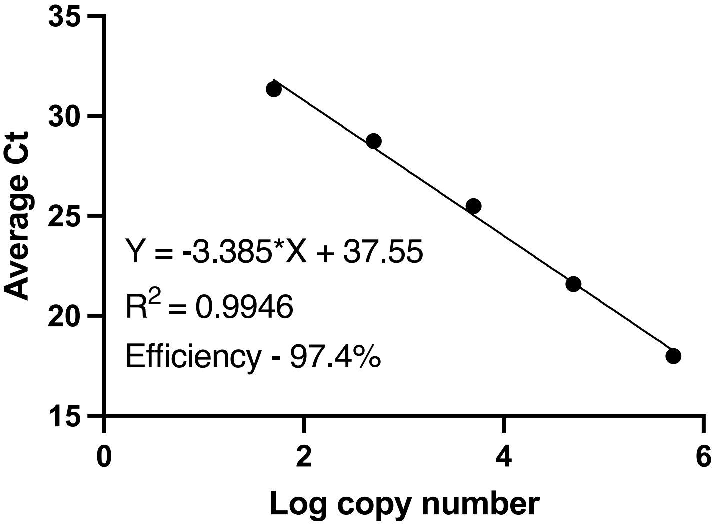

Figure S1: qPCR assay validation. Two primer sets were tested using known copy number of diluted DNA plasmid from which nucleocapsid RNA was transcribed. Efficiency and standard curve of the best primer set (used for this study) is presented here.

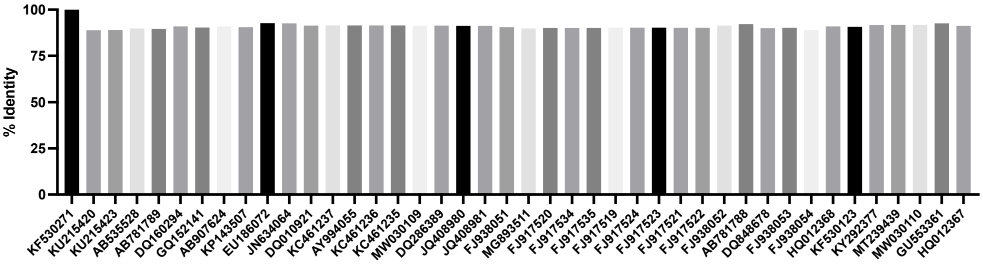

Figure S2: mRNA sequence comparison. Comparison of sequence identity between that chosen as template for vaccine (first sequence, KF530271) with ~40 additional published sequences. Genbank accessions used are shown across X axis.

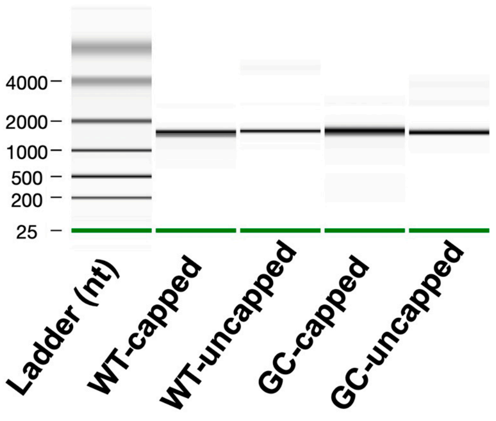

Figure S3. Bioanalyzer quality control from purified mRNA. Each construct was run for total RNA analysis with results for all four constructs shown here after column and cellulose purification (size: 1598nt).
